# Diet in Disease

**Published:** 1893-09-02

**Authors:** Ernest Hart

**Affiliations:** Bachelier-ès-Sciences-es-Lettres (rèstreint), formerly Student of the Faculty of Medicine of Paris, and of the London School of Medicine for Women


### Diet in Disease.

By Mrs. Ernest Hart, Bachelier-ès-Sciences-es-Lettres (rèstreint), formerly Student of the Faculty of Medicine of Paris, and of the London School of Medicine for Women.

### XXIX.—CHRONIC BRIGHT'S DISEASE.

*(Continued.)*

In many cases if the milk diet be persevered in continuously for several weeks, the albumen may entirely disappear from the urine, and if the kidney is not permanently or seriously damaged, it may not reappear even after a return to ordinary diet. If, however, there should be a reappearance of albumen, it may be necessary to return to the milk diet for a time. In the cases where there is incurable disease of the kidney, milk diet may improve the general condition of the health, but it cannot effect a cure. Some patients have been known to live on it for years, and have certainly suffered less discomfort, and have lived longer than if they had attempted to live on an ordinary diet. On leaving off the milk diet the return to an ordinary *régime* should be by cautious and slow degrees. A little arrowroot should first be mixed with the milk, then rice and tapioca milk puddings should be added, and finally a little fish, chicken, and cooked vegetables may be ventured on. Daily examinations of the urine should be made to see what influence the change of diet has on the excretion of the albumen, and if any particular articles of diet exercise a malign influence. If the albumen does not reappear, a light nutritious dietary can then be established. There are certain things which the albuminuric must avoid, and they are butcher's meat and alcohol.

There is a mistaken idea that because albumen is lost by the kidneys, there must be an excess of albumen in the food to make good this loss. But it must be remembered that the loss of albumen is not so serious and dangerous as the retention of urea and effete products, and that to prevent the formation of these is the first care in arranging the dietary of an albuminuric. Alcohol, except in those cases where there is an extreme weakness of the digestive powers, is forbidden. There is one thing, however, which an albuminuric may take in abundance, but which is often ignorantly withheld from him in the fear that it causes dropsy, and that is water. Water is an excellent diuretic in these cases. It dissolves out from the blood effete products, which are soluble, and it washes out the tubuli of the kidneys which are clogged with the *débris* of broken-down cells. Water should not be taken in excessive quantities at a time, but in small quantities at frequent intervals, and between meals rather than at meals. It will be found that it will diminish dropsy rather than provoke it, especially if the water taken is alkaline and slightly purgative in action. Professor Semmola, of Naples, recommends the following drink for the daily and habitual use of patients with Bright's disease: Sodium iodide, 15 grains; sodium phosphate, 30 grains; sodium chloride, 90 grains; drinking water, 36 ounces.

The study of the preparation of various dishes and soups which shall be at the same time nutritious and appetising, and yet not too stimulating and nitrogenous, becomes the duty of the housekeeper who has to cater for the victim of Bright's disease. These patients, particularly those who are suffering from the contracted-kidney form of disease, have feeble and capricious appetites, weak digestions, and they are often the subjects of constant nausea. To prepare the food so that it should be attractive, nutritious, and yet deficient in strong meats, will tax the art of the cook. Vegetable soups which may be varied from day to day, according to the vegetables in season, will be found to be most useful articles of diet in cases of chronic Bright's disease. I quote the following method of the preparation of these soups from Sir Henry Thompson's valuable and suggestive treatise on "Food and Feeding."

The following is a good recipe for a clear, purely vegetable stock: "Slice two carrots, two turnips, a head of celery, and two onions; put into a frying-pan with a few sweet herbs and half a pound of butter. Fry until well browned, then put them with three or four cloves, some salt and black pepper, into six pints of cold water in a saucepan; bring to the boil, and gently simmer for two or three hours, reducing to four pints, not less; strain off into a vessel, letting it stand for use. When required, pour off the clear liquor, leaving the deposit, and you will have a good vegetable stock. If it is to be used as a clear vegetable soup, heat, adding at the close two tablespoonfuls of cornflour previously mixed smooth in some of the liquor, and let the whole boil; if any scum arises, remove it. The cornflour gives to the decoction an agreeable body.

"To convert this into a meat *consommé* add after boiling, and just before serving, two full teaspoonfuls of the Liebig Company's Extract of Meat.

"Another mode of giving body when a soup *maigre* is not required is to make a decoction of beef bones without meat, which have been thoroughly broken and allowed to simmer gently at least six hours, then cooled and the fat removed. The result which is a strong jelly, can be warmed, strained clear through flannel, and used instead of water with which to make the vegetable soup as above directed; it adds substance and quality the animal matter takes the place of the cornflour employed for the preceding *soupe maigre.*

"Thickened vegetable soups may be made with these stocks, or with a weak meat stock, by rubbing in smooth, well-made *purées* of almost any vegetable matter. Those most commonly used are made from green peas, potato, carrot, turnip, artichoke, tomato, salsify, &c., or from dried vegetable products, as split peas, lentils, haricots, rice, arrowroot, semolina, &c."

Macaroni is also, as the same careful observer points out, an article of diet greatly neglected by the English, and would be a valuable one in the cases we are considering. The methods of cooking macaroni as recommended by Sir Henry Thompson, are, from personal experience, so excellent that I find it impossible to abridge them, and must ask permission to quote them in full.

"Put four ounces of good macaroni (Genoa or Naples), as little broken as possible, into a saucepan with three or four pints of boiling water. Boil ten minutes, not longer. Then pour off all the water, and place the macaroni in a stewpan with a pint of good and wellflavoured stock made from beef or veal, or both (or from a well-furnished stockpot), adding a saltspoon of salt and half that quantity of pepper, and let it simmer at the corner of the fire until the macaroni is tender; it is never to be soft and flabby. The time necessarily varies, according to the kind and size of the macaroni, *e.g.*, fifty or sixty minutes for the best Genoese, from twenty-five to thirty minutes for Neapolitan. Its condition, however, should be tested by trying a small piece. Most of the stock is absorbed by the macaroni by this time; but that which remains, probably a fourth part of the original quantity, may be strengthened, if necessary, by the third or the half of a teaspoonful of the genuine Liebig's Extract of Meat, and thickened by adding a little baked flour (baked quite brown), which is preferable for this purpose to the brown *roux* often used, which contains butter in a somewhat indigestible form. The above constitutes macaroni *au jus* in the simplest form.

"For those who can digest cheese and butter, an ounce of grated Parmesan, and, perhaps, half an ounce of good English cheese may be added gradually, stirring well during the latter half of the process, towards the end of which a little pat of butter may be added, with a sprinkle of Parmesan over the dish when filled, before serving. The macaroni ought now to "spin" well, that is, delicate threads should extend from one portion to another when moved. Lastly, hot tomato sauce may be poured over it, or be supplied separately, since some prefer the macaroni without this addition. Serve on a hot dish provided with a cover. It is now a dish of macaroni *à l'Italienne.*

"If there is only a weak stock, chiefly made from bones, &c., in the stock pot, use it, but add rather a larger portion of the Liebig's Extract. In such a case a little flour of lentils well boiled, to thicken the stock with, would be a suitable addition. The Liebig's Extract should never be added until the end of the process, and merely be well stirred in immediately after removing from the fire to serve.

"If, instead of stock, milk is used, an agreeable change may be made; and this form constitutes macaroni *au maigre*, the foregoing receipts being *au gras*. To prepare this, boil four ounces as before, ten minutes; drain and place in a stewpan with a pint of milk, simmering as above directed until sufficiently tender. Serve hot. Any milk remaining unabsorbed by the macaroni may be thickened with baked flour (white). Flavour with a little cinnamon or vanilla, or otherwise to taste, and sweeten with sugar or saccharin, if desired. For those who prefer a savoury dish, and can take cheese and butter, a tablespoonful of grated Parmesan and a small pat of butter should be gradually added, stirring it in during the latter part of the simmering process, according to the directions just given for macaroni *à l'Italienne.*"

These recipes are illustrative of the kind of diet which should be prepared for an albuminuric. Many of the recipes which have already been given for gout and rheumatism would be also applicable.

I must state, in conclusion, that the following list of dishes and articles is permissible:—All kinds of farinaceous food, rice, tapioca, arrowroot, hominy, oatmeal, cornflour, gruel, &c., cooked with milk or made into puddings. All kinds of well-cooked vegetables, avoiding in serious cases peas, beans, and lentils. Soups made of fish; *purées* of vegetables and thin bone stock; cocoa, coffee, and chocolate; cooked fruit, koumiss and junket, and fish and white meats in small quantity.

